# Adherence to treatment recommendations in older adults with vertigo, dizziness, and balance disorders: the MobilE-TRA 2 cohort study

**DOI:** 10.1007/s00415-026-14195-0

**Published:** 2026-10-08

**Authors:** Sophia P. Schulze, Benedict Katzenberger, Daniela Koller, Doreen Huppert, Eva Grill, Ralf Strobl

**Affiliations:** 1 Institute for Medical Information Processing, Biometry, and Epidemiology (IBE), LMU Medizin, LMU Munich, Marchioninistr. 15, 81377 Munich, Germany; 2 Pettenkofer School of Public Health, Elisabeth-Winterhalter-Weg 6, 81377 Munich, Germany; 3 Department of Orthopaedics and Trauma Surgery, Musculoskeletal University Center Munich (MUM), LMU University Hospital, LMU Medizin, LMU Munich, Munich, Germany; 4 German Center for Vertigo and Balance Disorders, LMU University Hospital, LMU Medizin, LMU Munich, Marchioninistr. 15, 81377 Munich, Germany; 5 Department of Neurology, LMU University Hospital, LMU Medizin, LMU Munich, Marchioninistr. 15, 81377 Munich, Germany

**Keywords:** Vertigo, Dizziness, Balance disorders, Patient adherence, Older adults, Therapy recommendations

## Abstract

**Background:**

Specific exercises and lifestyle-related recommendations are used to manage vertigo, dizziness, and balance disorders (VDB), but evidence on adherence and its determinants in older adults is limited. This study examined adherence changes and associations with condition-related and patient-related factors from 3 to 12 months after therapeutic recommendations.

**Methods:**

We used data from the MobilE-TRA 2 prospective cohort of adults aged ≥60 years treated for VDB at a tertiary referral centre in Germany. The participants were assessed at baseline and followed up after 3 and 12 months. Self-reported adherence was measured using a five-point Likert scale and dichotomised at predefined cut-offs. Perceived dizziness-related handicap was assessed using the Dizziness Handicap Inventory (DHI). Longitudinal associations were analysed using logistic generalised linear mixed-effects models with random intercepts.

**Results:**

Overall, 337 participants were included (53% women; mean age 71 years). In adjusted analyses, adherence to specific exercises was lower at 12 than at 3 months (odds ratio (OR) 0.28; 95% confidence interval (CI) 0.12–0.67). Higher baseline DHI scores were significantly associated with higher adherence to specific exercises (OR 1.03; 95% CI 1.00–1.06), but not with lifestyle-related recommendations (OR 1.00; 95% CI 0.98–1.02). Male gender was associated with lower adherence to lifestyle-related recommendations (OR 0.37; 95% CI 0.16–0.88), while higher self-efficacy was associated with higher adherence (OR 1.65; 95%CI 1.02–2.66).

**Conclusions:**

Adherence differed by recommendation type, participant characteristics and over time in older adults with VDB. These findings may inform strategies to support adherence in VDB beyond initial follow-up.

**Supplementary Information:**

The online version contains supplementary material available at https://doi.org/10.1007/s00415-026-14195-0.

## Introduction

Vertigo, dizziness, and balance disorders (VDB) are common conditions in older adults. Population-based studies estimate that 15–35% of adults experience VDB during their lifetime [1, 35]. Women are affected more often than men [35, 36], with data from Germany indicating a higher lifetime prevalence of moderate-to-severe VDB in women (36%) than in men (22%) [36]. The occurrence of VDB increases markedly with age; prevalence estimates reach approximately 30% among adults over 60 years and rise to nearly half among the oldest old, with reported rates of 45–51% in individuals aged 88–90 years [5, 20, 26]. VDB are associated with reduced mobility, limitations in activities of daily living, and an increased risk of falls [1, 12, 34].

Given this burden, effective management of VDB is important for maintaining mobility and functional independence. Treatment includes pharmacological and non-pharmacological approaches including specific exercises and lifestyle-related recommendations such as physical activity and weight-related behaviours [8, 13, 22]. Non-pharmacological interventions such as vestibular training and rehabilitation are often initiated under supervision of specialised health professionals. Yet, vestibular rehabilitation interventions require continued practice and implementation in everyday life to achieve long-term benefits [15, 22, 27]. Their effectiveness therefore largely depends on participants’ adherence.

Adherence is, as defined by the World Health Organization (WHO) framework, the extent to which a person’s behaviour corresponds with agreed recommendations from a healthcare provider [39]. The WHO framework conceptualises adherence as a multidimensional phenomenon influenced by five interacting domains: social and economic, health system-related, condition-related, therapy-related, and patient-related factors [39]. The present study focused on social and economic, condition-related, and patient-related factors for which corresponding baseline measures were available.

In line with this framework, previous research has examined a range of factors associated with adherence in rehabilitation settings [3, 16, 24]. However, much of this work has focused on musculoskeletal conditions such as osteoarthritis and chronic musculoskeletal pain, limiting its applicability to patients with VDB [16]. In physiotherapy research, barriers to adherence have been described across patient- and condition-related domains [24]. In the context of VDB, condition-related symptoms may influence adherence in different ways: mild or rapidly improving symptoms may reduce motivation to continue rehabilitation, whereas symptom provocation during exercises may act as a barrier to adherence [23, 27]. Evidence regarding demographic predictors, such as age and gender, on adherence remains inconsistent [16, 24].

Beyond demographic- and condition-related factors, patient-related characteristics may also influence adherence behaviour. Within this domain, concepts from the field of behavioural economics may be relevant for adherence. In particular, self-efficacy, time preferences, and health-related risk attitudes have been linked to the adoption of health-related behaviours [2, 11, 14]. Self-efficacy, an individual’s belief in their ability to perform specific behaviours [4], has been associated with adherence to rehabilitation and home-based exercise programmes in musculoskeletal settings, with lower self-efficacy identified as a barrier to adherence [3, 16, 24]. Time preferences describe the extent to which individuals value future outcomes relative to immediate rewards [17], while health-related risk attitudes describe an individual’s willingness to take risks in health-related contexts [14]. More present-oriented time preferences have been associated with lower engagement in preventive health behaviours and exercise [11, 31]. In addition, individual risk preferences have been associated with health-related behaviours such as smoking and alcohol consumption [2]. In patients with VDB, self-efficacy, risk attitudes, and time preferences have previously been investigated in relation to functioning [28]. However, their associations with adherence have received little attention: previous VDB studies on adherence have primarily examined clinical and symptom-related factors or barriers and facilitators of vestibular rehabilitation [23, 27]. Thus, whether self-efficacy, time preferences, and risk attitudes are associated with adherence to non-pharmacological recommendations in older adults with VDB remains unclear.

Consequently, longitudinal evidence on factors associated with adherence to non-pharmacological recommendations in older adults with VDB remains limited. The present study examines longitudinal adherence to non-pharmacological recommendations in older adults with VDB, distinguishing between specific exercises and lifestyle-related recommendations. In line with the WHO multidimensional adherence framework [39], we hypothesised that adherence would decrease over time and that adherence would be associated with factors across the social and economic, condition-related, and patient-related domains.

## Materials and methods

The study was reported in accordance with the STROBE guidelines [44].

### Study design, setting and data collection

MobilE-TRA 2 is a prospective observational cohort study of older adults with VDB conducted at University Hospital of Ludwig-Maximilians-Universität (LMU) with assessments at baseline and two follow-up time points: 3 months (FU1) and 12 months (FU2) after individual baseline assessment. The present analysis used data collected at the interdisciplinary outpatient clinic of the German Center for Vertigo and Balance Disorders (DSGZ), a tertiary referral centre for VDB that sees more than 3000 patients per year.

The participants were recruited consecutively during routine clinical consultations at the DSGZ. Eligible individuals were aged ≥60 years and attended their first interdisciplinary assessment for a VDB. All participants gave written informed consent. Ethical approval was granted by the Ethics Committee of the Medical Faculty of LMU Munich (#20–727), and the study was also conducted in accordance with the Declaration of Helsinki.

Baseline data were collected between December 2020 and June 2022 using standardised self-administered questionnaires administered at participants’ first presentation at the DSGZ, prior to the provision of any therapeutic recommendations. Selected clinical information was obtained from the DSGZ patient registry DizzyReg and linked to the study data using pseudonymised identifiers [21]. All participants underwent structured medical history taking and standardised neurological, ocular-motor, and neuro-otological examinations at the DSGZ. Additional investigations were performed as clinically indicated and included caloric testing, video head impulse testing, ocular and cervical vestibular-evoked myogenic potentials, posturography, audiometry, and auditory evoked potentials. Further imaging or assessment by other medical specialties was arranged where necessary. Results from these clinical and instrumental investigations were not included as predictors in the adherence models, which focused on factors assessed using standardised questionnaires administered to all participants. Follow-up data at FU1 and FU2 were collected using identical self-administered postal questionnaires. One reminder was sent in case of non-response; participants without a returned questionnaire were considered missing for the respective follow-up assessment and re-contacted in the next follow-up wave.

**Fig. 1:**
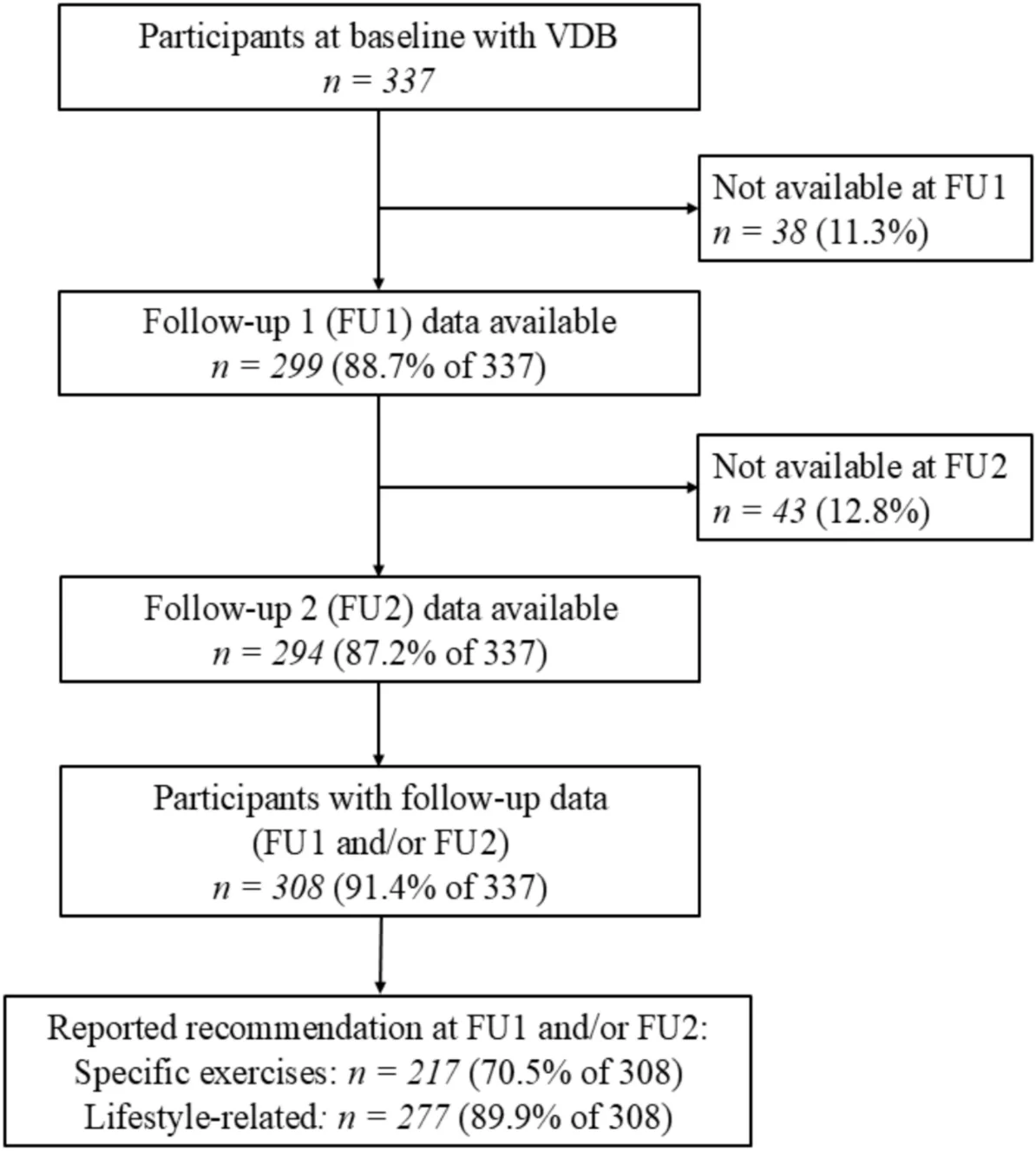
Flow of participants from baseline to follow-up and self-reported recommendation of specific exercises and lifestyle-related recommendations at FU1 and FU2

Participant flow from baseline to FU1 and FU2 and the number of participants reporting recommendations are shown in Fig. 1. Additional methodological details are reported in the published study protocol [29].

### Adherence

Adherence was defined according to the WHO as the degree to which a patient’s behaviour corresponds with therapeutic recommendations [39]. In the MobilE-TRA-2 study, adherence was assessed during FU1 and FU2 using self-reported implementation frequency measured on five-point Likert scale (0 = “never/almost never”, 1 = “occasionally”, 2 = “intermittently”, 3 = “often”, and 4 = “very often/always”), following a previously published adherence assessment method to enhance consistency and comparability with previous research [37].

Adherence was assessed for two non-pharmacological recommendation types used in the management of VDB: specific exercises, prescribed as part of the therapy of VDB, and lifestyle-related recommendations, addressing physical activity, general movement in everyday life (i.e., increasing routine day-to-day movement beyond structured physical activity), and weight control. Treatment recommendations were provided individually by the treating physician based on the clinical assessment. Participants could receive specific exercises, lifestyle-related recommendations, or both.

For the main analyses, adherence to both recommendation types was dichotomised a priori using specific cut-offs. While dichotomisation of ordinal adherence measures involves loss of information, this approach was chosen because a binary outcome allows an easy and clinically interpretable distinction between adherent and non-adherent participants, and increases model stability in the longitudinal mixed-effects analyses.

The adherence cut-offs were informed by the original operational approach of the adherence assessment [37]. Different cut-offs were applied to both recommendation types because they differed in therapeutic intent and expected implementation pattern. Adherence to specific exercises does not necessarily require daily or continuous performance [22], whereas adherence to lifestyle-related recommendations relies on frequent or consistent implementation. Accordingly, adherence to specific exercises was defined as a score of 2 or higher on the five-point Likert scale, and adherence to lifestyle-related recommendation as a score of 3 or higher. For descriptive analyses, the participants were classified as adherent if the respective criterion was met at least once at FU1 and/or FU2, and as non-adherent otherwise.

In order to capture the whole spectrum of adherence, participants additionally reported recommendations they had received from health professionals in a free-text field. These additional recommendations were reviewed and, where appropriate, assigned to the predefined categories. Adherence to specific exercises referred to self-reported implementation of exercises prescribed as part of VDB treatment. These may include exercise-based physiotherapeutic approaches such as balance, gait, or habituation exercises [13]. The adherence assessment did not distinguish between individual exercise types. Adherence to lifestyle-related recommendations was operationalised as a composite of physical activity, general movement, and weight control, reflecting closely related lifestyle-oriented recommendations.

Adherence analyses were restricted to follow-up assessments in which participants reported having received the respective recommendation and provided a valid self-reported implementation frequency at follow-up.

### Covariates

Covariates were categorised a priori in line with the WHO framework as social and economic, condition-related, and patient-related factors [39]. All covariates were assessed at baseline. A detailed description of all included covariates, including their operationalisation and sources, is provided in Online Resource 1, Supplementary Table S1.

Social and economic factors included age, gender, education and marital status. *Age* in years and *gender* (female/male) were self-reported. *Education* was classified according to the structure of the German educational system into low (primary/lower secondary education), middle (intermediate secondary education), and high (upper secondary or tertiary education). *Marital status* was categorised as single, married, divorced, or widowed.

Condition-related factors included perceived dizziness-related handicap, diagnostic subtype, and multimorbidity. *Perceived dizziness-related handicap* was used as an indicator of symptom-related impact, as both mild or rapidly improving symptoms and symptom provocation have been associated with lower adherence in patients with VDB [23, 27]. It was assessed using the Dizziness Handicap Inventory (DHI), a validated 25-item self-report questionnaire measuring functional, physical, and emotional impact in everyday life. Items were scored as 0 (“no”), 2 (“sometimes”), or 4 (“yes”), yielding a total score ranging from 0 to 100, with higher scores indicating greater perceived dizziness-related handicap [25]. For descriptive analyses, the DHI total score was additionally categorised using previously described severity categories: no handicap (≤ 14), mild (16–34), moderate (36–52), and severe (≥ 54) [30]. *Diagnostic subtypes* of VDB were based on the primary clinical diagnosis established at the DSGZ [28]. For the present analyses, diagnostic categories were categorised to reflect similarities in symptom course and therapeutic requirements. This categorisation was defined in collaboration with clinical experts at the DSGZ. Specifically, bilateral vestibulopathy and polyneuropathy were combined into the category “chronic vestibular disorders with sustained exercise demands”. The resulting diagnosis groups comprised chronic vestibular disorders with sustained exercise demands, benign paroxysmal positional vertigo (BPPV), persistent postural-perceptual dizziness (PPPD), Menière’s disease, multifactorial dizziness, vestibular migraine, orthostatic vertigo, central vestibular disorders, unilateral vestibulopathy, and a residual “other” category comprising less frequent diagnoses. The distribution of baseline diagnostic subtype groups is provided in Online Resource 1, Supplementary Table S2. *Multimorbidity* was defined as the presence of two or more self-reported chronic conditions in addition to VDB and included as a binary variable (yes/no) to reflect overall health burden potentially associated with adherence behaviour in older adults [3, 4]. The predefined list of self-reported comorbidities used to define multimorbidity is provided in Online Resource 1, Supplementary Table S3.

Patient-related factors included self-efficacy, health-related risk attitudes, and time preferences. Self-efficacy was assessed using the three-item General Self-Efficacy Short Scale [7]. The participants rated their agreement with three statements on confidence in handling difficult situations, managing problems independently, and solving complex tasks on a five-point Likert scale (1 = “doesn’t apply at all” to 5 = “applies completely”). A composite self-efficacy score was calculated as the mean of the three items (range: 1–5), with higher values indicating greater self-efficacy. *Health-related risk attitudes* were assessed using a single self-report item with an 11-point scale ranging from 0 (“not at all willing to take risks”) to 10 (“very willing to take risks”) [14]. *Time preferences* were assessed using two self-report items capturing future- and present-oriented preferences. Future-oriented preference was measured as willingness to wait for a larger future benefit rather than a smaller immediate benefit (11-point scale: 0 = “not willing at all” to 10 = “very willing”) [17]. Present-oriented preference was assessed using a five-point Likert scale (1 = “totally disagree” to 5 = “totally agree”) in response to the statement “I am only concerned about the present, because I trust that things will work themselves out in the future” [10].

### Statistical analyses

Descriptive analyses were used to characterise the study population and adherence at baseline and follow-up. The baseline characteristics were summarised using means and standard deviations for continuous variables and absolute and relative frequencies for categorical variables. Adherence at FU1 and FU2 was described using the original five-point Likert scale.

For selected descriptive analyses, baseline characteristics were compared between participants classified as adherent and non-adherent, separately for specific exercises and lifestyle-related recommendations. Continuous variables were compared using Wilcoxon rank-sum tests, and categorical variables using Pearson’s chi-square tests or Fisher’s exact tests when expected cell counts were small.

Longitudinal associations between baseline characteristics and adherence outcomes were analysed using generalised linear mixed-effects models (GLMMs) with a logit link. Separate models were fitted for adherence to specific exercises and adherence to lifestyle-related recommendations, with binary outcomes defined as described in the Adherence section. GLMMs allowed participants to contribute one or two follow-up observations, depending on data availability. A random intercept for participants was included to account for within-person correlation. Time was included as a fixed effect in all models. Random slopes for time were not included because only two repeated follow-up measurements were available per participant.

Covariate selection followed an a priori conceptual approach based on the WHO multidimensional adherence framework and was not driven by statistical significance in unadjusted analyses [39]. All covariates described in the Covariates section were included in the multivariable GLMMs according to predefined model specifications. Diagnostic subtype was not included in the GLMMs because several diagnostic groups were small and unevenly distributed across adherence categories, which would have led to unstable estimates and excessive model complexity relative to the number of outcome events. Continuous covariates were mean centred prior to inclusion to improve numerical stability and facilitate interpretation of the intercept.

For each outcome-specific GLMM, analyses were restricted to follow-up observations with complete information on the adherence outcome and all covariates included in the model; no imputation of missing data was performed. Consequently, analytic sample sizes differed between adherence outcomes, and participants could contribute data to one outcome-specific analysis but not to the other. To document the role of covariate adjustment, reduced GLMMs including time only and time plus social and economic and condition-related factors were additionally fitted and are provided in Online Resource 1, Supplementary Tables S4 and S5. These reduced models were not used for interpretation of the main findings.

Multicollinearity was assessed using the generalised variance inflation factor (GVIF) [18]. GVIF values greater than five were considered indicative of problematic multicollinearity. Exploratory interaction terms between time and perceived dizziness-related handicap were examined using likelihood-ratio tests (LRT) to evaluate potential effect modification.

All statistical tests were two-sided with a significance level of α = 0.05. Odds ratios (ORs) with 95% confidence intervals (CIs) were reported for fixed effects; the variance of the random intercept was reported to quantify between-person heterogeneity. Analyses were performed in R (version 4.4.3) [42]. The mixed-effects models were fitted using the lme4 package [6].

### Sensitivity analysis

Sensitivity analyses were performed to assess the robustness of the main findings and the impact of key analytical decisions. Alternative adherence cut-offs were applied to examine whether the results were sensitive to the dichotomisation of the Likert-scale responses. For specific exercises, a stricter cut-off was used, defining Likert scores of 3–4 as adherent and scores of 0–2 as non-adherent instead of the main cut-off of 2–4 versus 0–1. For lifestyle-related recommendations, a less stringent cut-off was applied, defining Likert scores of 2–4 as adherent and scores of 0–1 as non-adherent instead of the main cut-off of 3–4 versus 0–2. In addition, multimorbidity was redefined as three or more chronic conditions instead of two or more. All sensitivity analyses used the same multivariable model structure and complete-case approach as the main analyses. The reduced supplementary models were not repeated for the sensitivity analyses.

## Results

A total of 337 participants with VDB were included in the baseline survey. The mean age was 71 years (SD = 8). Overall, 53.1% (n = 179) were female. At FU1, follow-up questionnaires were returned by 88.7% of participants (n = 299), and at FU2 by 87.2% (n = 294). Documented reasons for non-participation at FU1 included declining any further follow-up participation (n = 6) and death (n = 1). At FU2, the study documentation recorded six participants declining participation and one death. A separate non-responder questionnaire was used to ascertain reasons for non-participation, but only three non-responders returned it. Consequently, reasons for non-participation were only partially available. Figure 1 illustrates participant flow from baseline to follow-up and the number of participants who reported having received a therapeutic recommendation at least once at FU1 and/or FU2.

The review of free-text “other recommendations” identified two additional participants who had received a recommendation for specific exercises, whereas no additional participants were identified for lifestyle-related recommendations.

For the analysis of adherence to specific exercises, 216 participants were included in the descriptive analyses. Based on the predefined cut-off (Likert scores 2–4), 79.6% (n = 172) were classified as adherent. The baseline characteristics of adherent and non-adherent participants are presented in Table 1. Adherent participants had higher baseline dizziness-related handicap compared with non-adherent participants, reflected in higher total DHI scores (44 vs. 37 points; *p* = 0.045) and higher physical DHI subscores (13 vs. 10 points; p = 0.005).

**Table 1:** Baseline characteristics by adherence to specific exercises (FU1 and/or FU2)

Characteristic	Overall N = 216^a^	Adherent N = 172^a^	Non-Adherent N = 44^a^	p-value^b^
Social and economic factors				
Age	71 (8)	71 (8)	72 (8)	0.574
Gender				0.742
Female	114	92 (81)	22 (19)	
Male	102	80 (78)	22 (22)	
Education				0.406
Low	84	63 (75)	21 (25)	
Middle	57	48 (84)	9 (16)	
High	73	59 (81)	14 (19)	
Marital status				0.263
Single	11	10 (91)	1 (9)	
Married	148	119 (80)	29 (20)	
Divorced	30	25 (83)	5 (17)	
Widowed	27	18 (67)	9 (33)	
Condition-related factors				
DHI^c^ overall score	43 (21)	44 (21)	37 (21)	0.045
DHI functional score	17 (10)	18 (9)	15 (11)	0.072
DHI physical score	12 (7)	13 (7)	10 (6)	0.005
DHI emotional score	13 (8)	13 (8)	12 (8)	0.128
Multimorbidity, yes	103	86 (83)	17 (17)	0.366
Patient-related factors				
Self-efficacy	3.95 (0.84)	3.97 (0.81)	3.90 (0.95)	0.900
Health-related risk attitudes	4.65 (2.21)	4.66 (2.19)	4.57 (2.31)	0.644
Future-oriented time preference	6.23 (2.11)	6.35 (2.10)	5.73 (2.11)	0.095
Present-oriented time preference	2.52 (1.02)	2.51 (1.01)	2.55 (1.06)	0.828

Continuous variables are reported as mean (SD). For categorical variables, the Overall column shows n, whereas adherence columns show n (%) with row percentages. Adherence was assessed only among participants reporting a recommendation for specific exercises. Adherent was defined as ≥1 Likert rating of 2–4 at FU1 or FU2; non-adherent as only ratings of 0–1 across available follow-ups. Participants without a valid adherence rating were excluded.

*DHI* dizziness handicap inventory, *FU1* first follow-up, *FU2* second follow-up, *N* number of participants, *SD* standard deviation

^a^Mean (SD); n (%)

^b^P-values: Wilcoxon rank-sum test for continuous variables and Pearson’s Chi-squared test for categorical variables

^c^Dizziness Handicap Inventory

For adherence to lifestyle-related recommendations, 270 participants were included in the descriptive analyses. Using the predefined cut-off (Likert scores 3–4), 80.4% (n = 217) were classified as adherent. Unadjusted comparisons of baseline characteristics between adherent and non-adherent participants are presented in Table 2. Adherent participants had higher baseline self-efficacy compared with non-adherent participants (4.03 vs. 3.71; p = 0.005).

**Table 2:** Baseline characteristics by adherence to lifestyle-related recommendations (FU1 and/or FU2)

Characteristic	Overall N = 270^a^	Adherent N = 217^a^	Non-adherent N = 53^a^	p value^b^
Social and economic factors				
Age	71 (8)	70 (7)	73 (10)	0.114
Gender				0.537
Female	143	117 (82)	26 (18)	
Male	127	100 (79)	27 (21)	
Education				0.670
Low	93	77 (83)	16 (17)	
Middle	71	55 (77)	16 (23)	
High	99	78 (79)	21 (21)	
Marital status				0.917
Single	16	13 (81)	3 (19)	
Married	182	148 (81)	34 (19)	
Divorced	39	31 (79)	8 (21)	
Widowed	33	25 (76)	8 (24)	
Condition-related factors				
DHI^c^ overall score	41 (21)	41 (22)	41 (21)	0.876
DHI functional score	17 (10)	17 (10)	17 (10)	0.713
DHI physical score	11 (7)	11 (7)	11 (6)	0.769
DHI emotional score	13 (8)	13 (8)	13 (9)	0.856
Multimorbidity, yes	139	114 (82)	25 (18)	0.484
Patient-related factors				
Self-efficacy	3.97 (0.83)	4.03 (0.83)	3.71 (0.81)	0.005
Health-related risk attitudes	4.62 (2.28)	4.66 (2.32)	4.46 (2.12)	0.662
Future-oriented time preference	6.21 (2.12)	6.33 (2.10)	5.70 (2.18)	0.064
Present-oriented time preference	2.52 (1.00)	2.55 (1.00)	2.37 (1.04)	0.229

Continuous variables are reported as mean (SD). For categorical variables, the Overall column shows n, whereas adherence columns show n (%) with row percentages. Adherence was assessed only among participants reporting a lifestyle-related recommendation. Adherent was defined as ≥1 Likert rating of 3–4 at FU1 or FU2; non-adherent as only ratings of 0–2 across available follow-ups. Participants without a valid adherence rating were excluded.

*DHI* dizziness handicap inventory, *FU1* first follow-up, *FU2* second follow-up, *N* number of participants, *SD* standard deviation

^a^Mean (SD); n (%)

^b^P-values: Wilcoxon rank-sum test for continuous variables and Pearson’s Chi-squared test for categorical variables

^c^Dizziness Handicap Inventory

**Fig. 2:**
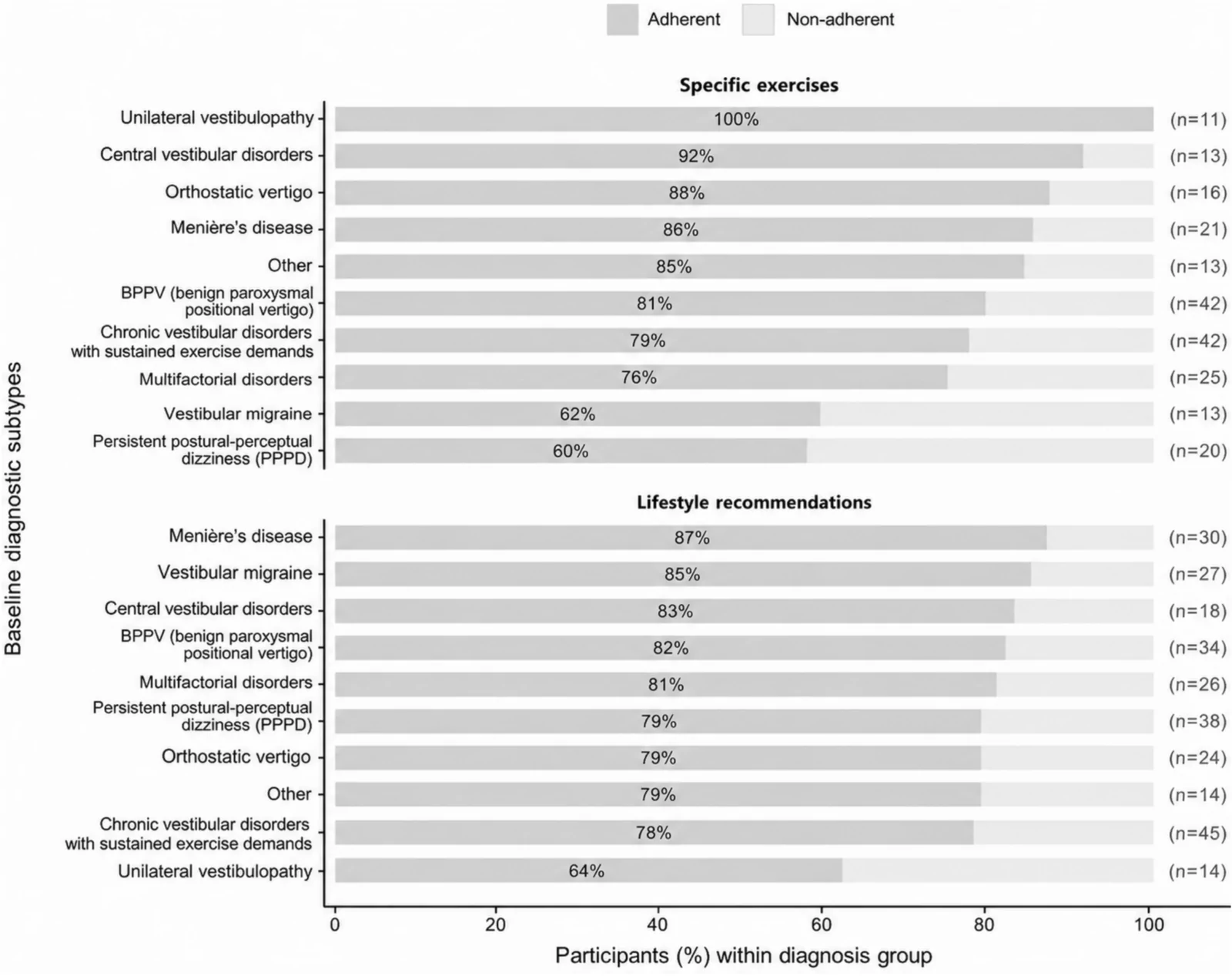
Adherence to therapeutic recommendations by baseline diagnostic subtype. One hundred percent stacked bar charts show the proportions of participants classified as adherent (dark grey) or -adherent (light grey) to specific exercises and lifestyle-related recommendations across baseline diagnostic subtypes. Participants were classified as adherent if they met the outcome-specific adherence criterion at least once at FU1 and/or FU2; all others were classified as non-adherent. Total numbers per diagnostic subtypes are shown to the right

At baseline, chronic vestibular disorders with sustained exercise demands were the most frequent diagnostic subtype (n = 57, 16.9%), followed by benign paroxysmal positional vertigo (BPPV; n = 48, 14.2%) and persistent postural-perceptual dizziness (PPPD; n = 48, 14.2%) (Online Resource 1, Supplementary Table S2). Adherence proportions by baseline diagnostic subtype are shown in Fig. 2, separately for specific exercises and lifestyle-related recommendations. Across diagnostic subtype, proportion adherent to specific exercises ranged from 60 to 100%. The lowest proportion of adherent participants was observed among those with PPPD (n = 20, 60%), while the highest proportion was observed among participants with unilateral vestibulopathy (n = 11, 100%). For lifestyle-related recommendations, adherence proportions ranged from 64 to 87% across diagnostic subtype, with the lowest adherence observed in unilateral vestibulopathy (n = 14, 64%) and the highest in Menière’s disease (n = 30, 87%). At baseline, the mean DHI score was 40.9 (SD = 21.4) among participants with available data (n = 312). Based on DHI severity categories, 58.4% (n = 182) had moderate or severe dizziness-related handicap, including 32.4% (n = 101) with severe handicap. 58.2% (n = 196) of participants were classified as multimorbid.

## Mixed model

Results of the generalised linear mixed-effects models are presented in Table 3.

**Table 3:** Associations of baseline factors and follow-up time with adherence to specific exercises and lifestyle-related recommendations: multivariable logistic mixed-effects models with participant-level random intercepts

Predictor	Specific exercises OR (95% CI) N = 303^a^	p value	Lifestyle-related recommendations OR (95% CI) N = 342^a^	p value
Time (12 months vs. 3 months)	0.28 (0.12–0.67)	0.004	0.70 (0.39–1.27)	0.242
Age	0.97 (0.90–1.04)	0.402	1.00 (0.95–1.05)	0.847
Gender (ref= Female)				
Male	0.83 (0.27–2.54)	0.739	0.37 (0.16–0.88)	0.025
DHI score	1.03 (1.00–1.06)	0.028	1.00 (0.98–1.02)	0.910
Multimorbidity (ref= No)				
Yes	0.61 (0.21–1.76)	0.358	1.04 (0.49–2.20)	0.928
Education (ref= Low)				
Middle	2.77 (0.69–11.17)	0.152	1.33 (0.52–3.39)	0.551
High	2.39 (0.62–9.24)	0.207	1.34 (0.54–3.34)	0.525
Marital status (ref= Single)				
Married	2.31 (0.24–22.17)	0.469	2.28 (0.50–10.50)	0.289
Divorced	1.46 (0.12–18.36)	0.768	1.16 (0.20–6.88)	0.867
Widowed	1.24 (0.08–18.24)	0.877	1.90 (0.29–12.45)	0.505
Self-efficacy	1.24 (0.63–2.43)	0.533	1.65 (1.02–2.66)	0.042
Health-related risk attitudes	1.07 (0.84–1.36)	0.604	0.89 (0.75–1.06)	0.182
Future-oriented time preference	1.30 (0.99–1.72)	0.061	1.16 (0.96–1.41)	0.120
Present-oriented time preference	0.95 (0.55–1.62)	0.844	0.85 (0.58–1.25)	0.401
Between-person variance^b^	5.033		2.076	

ORs and 95% CIs are shown. All predictors except follow-up time were assessed at baseline. Reference categories were female gender, no multimorbidity, low education, and single marital status. Continuous covariates were mean-centred. ORs > 1 indicate higher odds of adherence.

*CI* confidence interval, *DHI* dizziness handicap inventory, *OR* odds ratio, ref reference category

^a^Total number of follow-up observations included in each model. For specific exercises, 172 participants contributed 303 observations (41 participants with one and 131 with two follow-up assessments). For lifestyle-related recommendations, 204 participants contributed 342 observations (66 participants with one and 138 with two follow-up assessments)

^b^Variance component of the participant-level random intercept

For adherence to specific exercises, adherence decreased significantly from FU1 to FU2 (OR 0.28; 95% CI 0.12–0.67; p = 0.004). Higher dizziness-related handicap was significantly associated with higher adherence (OR 1.03; 95% CI 1.00–1.06; p = 0.028).

Adherence to lifestyle-related recommendations did not differ significantly between FU1 and FU2 (OR 0.70; 95% CI 0.39–1.27; p = 0.242). Male gender was associated with lower odds of adherence (OR 0.37; 95% CI 0.16–0.88; p = 0.025), whereas higher self-efficacy was associated with higher odds of adherence (OR 1.65; 95% CI 1.02–2.66; p = 0.042).

Reduced GLMMs are provided in Online Resource 1, Supplementary Tables S4 and S5. These models document how estimates changed after covariate adjustment but were not used for interpretation of the main findings.

No evidence of relevant multicollinearity was observed (all GVIF < 5). Exploratory analysis did not reveal interaction between time and dizziness-related handicap, indicating that the association between baseline dizziness-related handicap and adherence did not differ between FU1 and FU2.

### Sensitivity analysis

Sensitivity analyses are provided in Online Resource 1, Supplementary Tables S6 and S7. For adherence to specific exercises, redefining multimorbidity as three or more chronic conditions yielded similar findings, with lower odds of adherence at FU2 and higher odds of adherence among participants with higher baseline DHI scores. Applying the stricter adherence cut-off for specific exercises (Likert scores 3–4 versus 0–2) led to some differences in the estimates. In this analysis, multimorbidity defined as two or more chronic conditions was associated with lower odds of adherence (OR 0.22; 95% CI 0.06–0.81), and higher baseline self-efficacy was associated with higher odds of adherence (OR 3.01; 95%CI 1.20–7.59). Associations with follow-up time and baseline DHI were no longer statistically significant.

For adherence to lifestyle-related recommendations, redefining multimorbidity as three or more chronic conditions yielded estimates similar to those of the main analysis, with male gender and higher baseline self-efficacy remaining associated with adherence. Applying the less stringent adherence cut-off for lifestyle-related recommendations (Likert scores 2–4 versus 0–1) resulted in no statistically significant associations between covariates and adherence. Confidence intervals were substantially wider, and the random-intercept variance increased markedly. Therefore, results from this specification were interpreted with caution.

## Discussion

In this prospective observational cohort study of older adults with VDB conducted at a specialised tertiary care centre, adherence to non-pharmacological treatment recommendations after 3 and 12 months was high and did not differ relevantly between specific exercises and lifestyle-related recommendations. We found that adherence to specific exercises decreased from 3 to 12 months after the initial assessment and was higher in participants with greater baseline dizziness-related handicap. Adherence to lifestyle-related recommendations remained stable over time and was lower in men and higher in participants with high self-efficacy.

### Interpretation

Adherence to specific exercises decreased between 3 and 12 months after initial assessment at the tertiary care centre. In patients with VDB, several barriers may contribute to difficulties in maintaining vestibular exercises over time. These include symptom provocation, time constraints, associated impairments, and insufficient guidance or feedback [27]. Moreover, individuals with vestibular disorders report higher fear-avoidance beliefs, greater perceived disability, and lower confidence during balance activities than healthy adults [43]. Evidence from customised vestibular rehabilitation further indicates that adherence may vary according to vestibulopathy category and symptom severity, with mild or rapidly improving symptoms being associated with lower adherence in some vestibulopathy subgroups and balance impairments posing additional challenges to participation [23]. In this context, the decrease in adherence observed in our study should not necessarily be interpreted as failure to maintain the recommended exercises. It may partly reflect a reduced need for continued exercise as symptoms improve or resolve over the course of treatment. Thus, lower adherence over time may have different clinical implications depending on the course of symptoms. More broadly, difficulties in maintaining adherence to self-managed home-based exercise have also been reported across physiotherapy and chronic disease populations [16, 24, 38]. Adherence to lifestyle-related recommendations, in contrast, did not change significantly between FU1 and FU2. Evidence specifically addressing adherence to lifestyle-related recommendations in patients with VDB is limited, and the present study did not assess reasons for the observed stability.

In our study, higher perceived dizziness-related handicap was associated with higher adherence to specific exercises. Previous research in patients with VDB has also indicated that symptom-related factors may influence adherence, although findings differ depending on the clinical context [23, 27]. For example, mild or rapidly improving symptoms have been associated with lower adherence in some vestibulopathy subgroups [23], whereas symptom provocation during exercises has been identified as a barrier to adherence [27]. Evidence from other chronic conditions is heterogeneous [38], suggesting that the association between symptom burden and adherence may be context-specific.[41]. As these assessments were performed in our study according to clinical indication rather than uniformly across the cohort, their inclusion in the models would have restricted the analyses to a clinically selected subgroup. In our study, we found that higher self-efficacy was associated with higher adherence to lifestyle-related recommendations. This is consistent with systematic reviews in physiotherapy, where low self-efficacy is described as a barrier to adherence [16, 24], and with broader behavioural evidence linking self-efficacy to health-related behaviour [40]. Self-efficacy may support adherence because it reflects participants’ perceived ability to implement and maintain health-related behaviours in everyday life [4]. This may be particularly relevant in VDB, where patients need to manage symptoms such as dizziness and unsteadiness while implementing recommendations in everyday life. Previous longitudinal evidence from the MobilE-TRA2 cohort also identified self-efficacy as a predictor of functioning in older participants with VDB after specialised care [28]. Although functioning and adherence are conceptually distinct, both may depend on participants’ perceived ability to manage VDB-related challenges and sustain recommended behaviours.

In our study neither time preferences nor health-related risk attitudes were statistically significantly associated with adherence. Although associations with health-related behaviours have been reported in other contexts [2, 11, 31, 33], our findings do not provide evidence for corresponding associations with adherence to non-pharmacological recommendations in this VDB cohort.

In our study, male gender was associated with lower adherence to lifestyle-related recommendations. Evidence specifically addressing gender differences in adherence to lifestyle-related recommendations in patients with VDB is lacking. In broader lifestyle intervention research, however, the association between gender and adherence has been inconsistent, with male gender associated with both lower and higher adherence depending on the intervention and adherence measure [32]. Similarly, a systematic review of home-based exercise therapy in patients with chronic diseases found no evidence that gender predicted exercise adherence [38]. Thus, the lower adherence among men observed in our study does not appear to reflect a consistent gender-related pattern across non-pharmacological health behaviours. As the present study did not assess reasons for non-adherence, the mechanisms underlying this association remain unclear. Future studies should investigate whether gender differences vary according to the type of lifestyle recommendation and explore potential barriers to their implementation.

### Strengths

Strengths of this study include its prospective longitudinal design, the clinical cohort of 337 older adults with VDB, and the high follow-up response rates at both 3 and 12 months. The repeated follow-up assessments allowed adherence to different types of non-pharmacological recommendations to be examined over time. Participants underwent standardised interdisciplinary assessment and comprehensive diagnostic work-up at a specialised tertiary referral centre, providing a well-characterised clinical cohort. In addition, the study considered patient-related factors that have received limited attention in adherence research in VDB, including self-efficacy, time preferences, and health-related risk attitudes.

### Limitations

Several limitations should be considered when interpreting our findings. First, receipt and implementation of treatment recommendations were self-reported and may therefore be affected by recall and social desirability bias. Standardised self-administered questionnaires were used to minimise interviewer-related influences, and free-text responses were additionally reviewed to identify recommendations not captured by the predefined categories. Nevertheless, the reported adherence levels should be interpreted as perceived implementation of recommendations rather than as objectively measured adherence.

Second, adherence measurement has not yet been standardised for VDB, and the category “specific exercises” may have comprised different types of exercises across participants and diagnostic subtypes. Moreover, adherence was dichotomised for the main analyses, resulting in a loss of information and requiring outcome-specific cut-offs. We therefore examined alternative cut-offs in sensitivity analyses. As some associations changed under these alternative definitions, particularly for specific exercises, individual effect estimates should be interpreted with caution. Our findings should therefore be interpreted in the context of the broader lack of standardised adherence measurement in home-based rehabilitation [9, 19].

Third, diagnostic subtype could not be included in the multivariable models because several diagnostic groups were small and unevenly distributed across adherence categories, which would have resulted in unstable estimates. Given differences in therapeutic requirements across VDB diagnoses, residual confounding by diagnostic subtype therefore cannot be excluded. To provide transparency regarding potential diagnosis-specific differences, adherence was additionally reported descriptively by diagnostic subtype. Larger studies are needed to investigate diagnosis-specific determinants of adherence in multivariable analyses.

Finally, additional physician or therapist contacts and changes in treatment recommendations between follow-up assessments were not captured. Participants with persistent symptoms or insufficient treatment response may have been more likely to seek additional care and receive modified recommendations or professional support. Consequently, changes in adherence over time cannot be attributed exclusively to the recommendations provided at the initial assessment. Future longitudinal studies should therefore capture healthcare contacts and changes in treatment recommendations during follow-up.

Furthermore, the present study did not assess health system-related or therapy-related factors included in the WHO multidimensional adherence framework. Therefore, the potential contribution of these domains to adherence could not be evaluated.

### Transferability

The transferability of these findings to general practice may be limited by differences in patient case-mix and care delivery. Patients attending a tertiary care centre are likely to have more persistent, severe, or complex vestibular disorders and may receive more individualised exercise recommendations than patients managed in primary care. Consequently, absolute adherence levels and associations with potential determinants may differ between settings. Replication in primary-care and other clinical populations is needed to determine whether the observed longitudinal patterns and associated factors are generalisable beyond specialised tertiary care.

In addition, recruitment took place during the SARS-CoV-2 pandemic, when access to healthcare and referral pathways were temporarily altered. This may have resulted in a more selected clinical population, potentially including patients with more severe or persistent symptoms. The observed adherence levels and associations should therefore be interpreted in light of these pandemic-related changes in healthcare access and patient selection and may not be fully transferable to patients treated under non-pandemic conditions.

## Conclusion

In this prospective observational cohort study of older adults with VDB, adherence to specific exercises decreased between 3 and 12 months, whereas adherence to lifestyle-related recommendations remained stable. Greater baseline dizziness-related handicap was associated with higher adherence to specific exercises, while higher self-efficacy was associated with higher adherence to lifestyle-related recommendations and male gender with lower adherence. The differing patterns observed for the two adherence outcomes suggest that recommendation type should be considered when investigating adherence in VDB. Further studies are needed to examine reasons for non-adherence and to confirm these findings in other clinical settings.

## Supplementary Information

Below is the link to the electronic supplementary material.

Supplementary file1 (DOCX 53 KB)

## Data Availability

The data and analysis code are available from the corresponding author upon reasonable request, subject to applicable data protection and ethical restrictions.
