# Supplementary material for: Adherence to treatment recommendations in older adults with vertigo, dizziness, and balance disorders: the MobilE-TRA 2 cohort study
Source: J Neurol. 2026 Oct 8;273(10):649. doi: 10.1007/s00415-026-14195-0 (PMC13645870; doi:10.1007/s00415-026-14195-0)
Supplement: Supplementary file 1 [file 415_2026_14195_MOESM1_ESM.docx]

**Supplementary tables for “Adherence to Treatment Recommendations in Older Adults with Vertigo, Dizziness, and Balance Disorders: The MobilE-TRA 2 Cohort Study”**

**Journal of Neurology**

Sophia-P. Schulze^1,2^, Benedict Katzenberger^1,2^, Daniela Koller^1,3^, Doreen Huppert^4,5^

Eva Grill^1,4^, Ralf Strobl^1,4^

^1^Institute for Medical Information Processing, Biometry and Epidemiology (IBE), LMU Medizin, LMU Munich, Marchioninistr. 15, 81377 Munich, Germany

^2^Pettenkofer School of Public Health, Elisabeth-Winterhalter-Weg 6, 81377 Munich, Germany

^3^Department of Orthopaedics and Trauma Surgery, Musculoskeletal University Center Munich (MUM), LMU University Hospital, LMU Medizin, LMU Munich, Munich, Germany

^4^German Center for Vertigo and Balance Disorders, LMU University Hospital, LMU Medizin, LMU Munich, Marchioninistr. 15, 81377 Munich, Germany

^5^Department of Neurology, LMU University Hospital, LMU Medizin, LMU Munich, Marchioninistr. 15, 81377 Munich, Germany

**Correspondence:**
Dr. Ralf Strobl
German Center for Vertigo and Balance Disorders, LMU University Hospital, LMU Medizin, LMU Munich, Marchioninistr. 15, 81377 Munich, Germany

E-mail: [ralf.strobl@med.uni-muenchen.de](mailto:ralf.strobl@med.uni-muenchen.de)

**Supplementary Table S1.** Overview of covariates with operationalisation and literature-based rationale

The selection of covariates was guided by the WHO multidimensional framework of adherence [3]. Variables were selected a priori as baseline factors potentially associated with adherence in older adults with VDB and were grouped into social and economic, condition-related, and patient-related domains. References provide the empirical or theoretical rationale for each variable.

| Variable | Operationalisation / Coding | Included GLMM | Literature-based rationale (with references) |
| --- | --- | --- | --- |
| **SOCIAL AND ECONOMIC FACTORS** | | | |
| Age | Continuous (years), centred at mean | Yes | Core demographic characteristic commonly examined in adherence research; age has been reported as an inconsistent predictor of adherence in physiotherapy and rehabilitation settings [1–3]. |
| Gender | Binary (female/male; female reference) | Yes | Standard demographic variable frequently considered in adherence research and included in line with the WHO multidimensional framework of adherence [1–3]. |
| Education | Categorised into low (primary/lower secondary education), middle (intermediate secondary education), and high (upper secondary or tertiary education) | Yes | Indicator of socioeconomic position; social and economic factors are a core domain within the WHO multidimensional framework of adherence [3]. |
| Marital status | Single / Married / Divorced / Widowed | Yes | Captures social and household context; social support and social circumstances have been discussed as factors relevant to adherence in chronic conditions [1, 3, 4]. |
| **CONDITION-RELATED FACTORS** | | | |
| DHI total score | Continuous (0–100), centred; severity categories used descriptively (no handicap <16; mild 16–34; moderate 35–52; severe ≥54) | Yes | Indicator of perceived dizziness-related handicap and symptom-related impact; symptom-related factors have been examined in relation to adherence and rehabilitation engagement in VDB, and the DHI is a validated measure of dizziness-related impact [5–7]. |
| Diagnostic subtype | Baseline primary clinical diagnosis (DSGZ), analytically grouped; details in Supplementary Table S2 | No (descriptive only) | VDB diagnoses differ in symptom course and therapeutic requirements; diagnostic classification was based on the MobilE-TRA 2 framework and the DSGZ registry structure [8, 9]. |
| Multimorbidity | ≥2 self-reported chronic conditions in addition to VDB (yes/no); details in Supplementary Table S3 | Yes | Indicator of overall health burden and competing treatment demands; multimorbidity has been examined as a potential determinant of adherence in chronic conditions [1, 3, 10]. |
| **PATIENT-RELATED FACTORS** | | | |
| Self-efficacy (German General Self-Efficacy Short Scale [Allgemeine Selbstwirksamkeit Kurzskala, ASKU]) | Mean of 3 ASKU items (range 1–5); higher values indicate greater self-efficacy | Yes | General self-efficacy was assessed using the validated German General Self-Efficacy Short Scale (ASKU) [11]. Self-efficacy reflects perceived capability to manage challenging situations and has been associated with adherence to home-based exercise and with functioning in older adults with VDB [2, 8, 12–14]. |
| Health-related risk attitudes | Single-item scale (0–10; higher = greater willingness to take risks) | Yes | Risk attitudes capture willingness to accept uncertainty and have been examined in relation to health behaviours and adherence-related decisions [15–17]. |
| Future-oriented time preference | 0–10 scale (willingness to wait for larger future benefit) | Yes | Future-oriented time preference reflects valuation of delayed benefits. The measure was based on willingness to wait for a larger future benefit [18] and has been linked to health-maintenance behaviours and adherence-related decisions [17, 19, 20]. |
| Present-oriented time preference | 5-point Likert item (agreement with present-focused statement) | Yes | Present-oriented preferences may compete with sustained engagement in behaviours that yield longer-term benefits; the item has been used in research on time preference and health behaviour [20, 21]. |

Abbreviations: ASKU General Self-Efficacy Short Scale; DHI Dizziness Handicap Inventory; DSGZ German Center for Vertigo and Balance Disorders; GLMM generalised linear mixed-effects model; VDB vertigo, dizziness, and balance disorders; WHO World Health Organization.

**References**

1. Jack K, McLean SM, Moffett JK, Gardiner E (2010) Barriers to treatment adherence in physiotherapy outpatient clinics: a systematic review. Man Ther 15:220–228. https://doi.org/10.1016/j.math.2009.12.004

2. Essery R, Geraghty AW, Kirby S, Yardley L (2017) Predictors of adherence to home-based physical therapies: a systematic review. Disabil Rehabil 39:519–534. https://doi.org/10.3109/09638288.2016.1153160

3. Sabaté E (2003) Adherence to long-term therapies: evidence for action. World Health Organization, Geneva

4. Vermeire E, Hearnshaw H, Van Royen P, Denekens J (2001) Patient adherence to treatment: three decades of research. A comprehensive review. J Clin Pharm Ther 26:331–342. https://doi.org/10.1046/j.1365-2710.2001.00363.x

5. Jacobson GP, Newman CW (1990) The development of the Dizziness Handicap Inventory. Arch Otolaryngol Head Neck Surg 116:424–427. https://doi.org/10.1001/archotol.1990.01870040046011

6. Kalderon L, Kaplan A, Wolfovitz A, Levy-Tzedek S, Gimmon Y (2024) Barriers and Facilitators of Vestibular Rehabilitation: Patients and Physiotherapists' Perspectives. J Neurol Phys Ther 48:140–150. https://doi.org/10.1097/NPT.0000000000000470

7. Im YH, Lee HJ, Jeon EJ (2025) Adherence to customized vestibular rehabilitation therapy: influencing factors and clinical implications in vestibulopathy. Front Neurol 16:1538989. https://doi.org/10.3389/fneur.2025.1538989

8. Katzenberger B, Fuchs S, Schwettmann L, Strobl R, Hauser A, Koller D, Grill E (2023) Association of self-efficacy, risk attitudes, and time preferences with functioning in older patients with vertigo, dizziness, and balance disorders in a tertiary care setting—Results from the MobilE-TRA2 cohort. Front Neurol 14:1316081. https://doi.org/10.3389/fneur.2023.1316081

9. Katzenberger B, Schwettmann L, Weigl M, Paulus A, Pedron S, Fuchs S, Koller D, Grill E (2021) Behavioural and patient-individual determinants of quality of life, functioning and physical activity in older adults (MobilE-TRA 2): study protocol of an observational cohort study in a tertiary care setting. BMJ Open 11:e051915. https://doi.org/10.1136/bmjopen-2021-051915

10. Ricke E, Dijkstra A, Bakker EW (2023) Prognostic factors of adherence to home-based exercise therapy in patients with chronic diseases: a systematic review and meta-analysis. Front Sports Act Living 5:1035023. https://doi.org/10.3389/fspor.2023.1035023

11. Beierlein C, Kemper CJ, Kovaleva A, Rammstedt B (2013) Short Scale for Measuring General Self-efficacy Beliefs (ASKU). Methods Data Anal 7:251–278. https://doi.org/10.12758/mda.2013.014

12. Bandura A (1977) Self-efficacy: toward a unifying theory of behavioral change. Psychol Rev 84:191–215. https://doi.org/10.1037/0033-295X.84.2.191

13. Argent R, Daly A, Caulfield B (2018) Patient Involvement With Home-Based Exercise Programs: Can Connected Health Interventions Influence Adherence? JMIR Mhealth Uhealth 6:e47. https://doi.org/10.2196/mhealth.8518

14. Náfrádi L, Nakamoto K, Schulz PJ (2017) Is patient empowerment the key to promote adherence? A systematic review of the relationship between self-efficacy, health locus of control and medication adherence. PLoS One 12:e0186458. https://doi.org/10.1371/journal.pone.0186458

15. Anderson LR, Mellor JM (2008) Predicting health behaviors with an experimental measure of risk preference. J Health Econ 27:1260–1274. https://doi.org/10.1016/j.jhealeco.2008.05.011

16. Dohmen T, Falk A, Huffman D, Sunde U, Schupp J, Wagner GG (2011) Individual risk attitudes: measurement, determinants, and behavioral consequences. J Eur Econ Assoc 9:522–550. https://doi.org/10.1111/j.1542-4774.2011.01015.x

17. van der Pol M, Hennessy D, Manns B (2017) The role of time and risk preferences in adherence to physician advice on health behavior change. Eur J Health Econ 18:373–386. https://doi.org/10.1007/s10198-016-0800-7

18. Falk A, Becker A, Dohmen T, Enke B, Huffman D, Sunde U (2018) Global evidence on economic preferences. Q J Econ 133:1645–1692. https://doi.org/10.1093/qje/qjy013

19. Bradford WD (2010) The association between individual time preferences and health maintenance habits. Med Decis Making 30:99–112. https://doi.org/10.1177/0272989X09342276

20. Lawless L, Drichoutis AC, Nayga RM (2013) Time preferences and health behaviour: a review. Agric Food Econ 1:17. https://doi.org/10.1186/2193-7532-1-17

21. Borghans L, Golsteyn BHH (2006) Time discounting and the body mass index: evidence from the Netherlands. Econ Hum Biol 4:39–61. https://doi.org/10.1016/j.ehb.2005.10.001

**Supplementary Table S2.** Distribution of baseline diagnostic subtype groups among participants at baseline, FU1, and FU2

Diagnostic subtypes of VDB were based on the primary clinical diagnosis established at the DSGZ. For descriptive presentation, diagnostic categories were grouped to reflect similarities in symptom course and therapeutic requirements. This categorization was defined in collaboration with clinical experts at the DSGZ. Specifically, bilateral vestibulopathy and polyneuropathy were combined into the category “chronic vestibular disorders with sustained exercise demands”. Less frequent diagnoses were grouped into a residual “Other” category.

| **Diagnosis group** | **Baseline n (%)**  **(N = 337)** | **FU1 n (%)**  **(N = 299)** | **FU2 n (%)**  **(N = 294)** |
| --- | --- | --- | --- |
| Chronic vestibular disorders with sustained exercise demands | 57 (16.9%) | 51 (17.1%) | 49 (16.7%) |
| BPPV | 48 (14.2%) | 44 (14.7%) | 43 (14.6%) |
| Persistent Postural-Perceptual Dizziness (PPPD) | 48 (14.2%) | 39 (13.0%) | 38 (12.9%) |
| Menière’s disease | 33 (9.8%) | 31 (10.4%) | 31 (10.5%) |
| Multifactorial dizziness | 30 (8.9%) | 25 (8.4%) | 24 (8.2%) |
| Vestibular migraine | 29 (8.6%) | 29 (9.7%) | 27 (9.2%) |
| Orthostatic vertigo | 29 (8.6%) | 25 (8.4%) | 26 (8.8%) |
| Central vestibular disorders | 22 (6.5%) | 19 (6.4%) | 21 (7.1%) |
| Unilateral vestibulopathy | 18 (5.3%) | 16 (5.4%) | 15 (5.1%) |
| Other | 23 (6.8%) | 20 (6.7%) | 20 (6.8%) |

Note: Diagnosis groups were assigned at baseline. Percentages for FU1 and FU2 refer to participants with available follow-up data at the respective assessment. BPPV benign paroxysmal positional vertigo, DSGZ German Center for Vertigo and Balance Disorders, FU1 first follow-up, FU2 second follow-up, PPPD persistent postural-perceptual dizziness, VDB vertigo, dizziness, and balance disorders.

**Supplementary Table S3.** Predefined list of self-reported comorbidities in the DizzyReg patient registry

Comorbidities were captured via patient self-report at baseline and follow-up using a predefined list of conditions. For the present analyses, baseline information was used to define multimorbidity as the presence of two or more chronic conditions in addition to VDB:

- Angina pectoris, coronary sclerosis

- Asthma

- Cardiac arrhythmia

- Cardiac insufficiency

- Chronic bronchitis

- Chronic obstructive pulmonary disease or emphysema

- Hay fever

- Hypertension

- Inflammatory joint disease

- Kidney disease

- Liver disease (e.g. cirrhosis)

- Neurological disease (multiple sclerosis, epilepsy)

- Other disease

**Supplementary Table S4.** Associations of baseline factors and follow-up time with adherence to specific exercises: sequential logistic mixed-effects models with participant-level random intercepts

| **Predictor** | **Model 1**  **OR (95% CI)** | **p** | **Model 2**  **OR (95% CI)** | **p** | **Model 3**  **OR (95% CI)** | **p** |
| --- | --- | --- | --- | --- | --- | --- |
| N (observations)^a^ | 316 |  | 316 |  | 303 |  |
| Between-person variance^b^ | 3.526 |  | 3.416 |  | 5.033 |  |
| Time (12 months vs. 3 months) | 0.40 (0.20–0.79) | 0.009 | 0.38 (0.19–0.77) | 0.007 | 0.28 (0.12–0.67) | 0.004 |
| Age | – | – | 0.96 (0.90–1.03) | 0.242 | 0.97 (0.90–1.04) | 0.402 |
| Gender (ref = Female) |  |  |  |  |  |  |
| Male | – | – | 0.96 (0.37–2.47) | 0.932 | 0.83 (0.27–2.54) | 0.739 |
| DHI score | – | – | 1.02 (1.00–1.05) | 0.040 | 1.03 (1.00–1.06) | 0.028 |
| Multimorbidity (ref = No) | |  |  |  |  |  |
| Yes | – | – | 0.83 (0.34–2.02) | 0.687 | 0.61 (0.21–1.76) | 0.358 |
| Education (ref = Low) |  |  |  |  |  |  |
| Middle | – | – | 2.61 (0.83–8.19) | 0.100 | 2.77 (0.69–11.17) | 0.152 |
| High | – | – | 2.43 (0.83–7.13) | 0.107 | 2.39 (0.62–9.24) | 0.207 |
| Marital status (ref = Single) | |  |  |  |  |  |
| Married | – | – | 1.50 (0.21–10.54) | 0.683 | 2.31 (0.24–22.17) | 0.469 |
| Divorced | – | – | 1.50 (0.17–13.23) | 0.713 | 1.46 (0.12–18.36) | 0.768 |
| Widowed | – | – | 1.16 (0.11–11.78) | 0.900 | 1.24 (0.08–18.24) | 0.877 |
| Self-efficacy | – | – | – | – | 1.24 (0.63–2.43) | 0.533 |
| Health-related risk attitudes | – | – | – | – | 1.07 (0.84–1.36) | 0.604 |
| Future-oriented time preference | – | – | – | – | 1.30 (0.99–1.72) | 0.061 |
| Present-oriented time preference | – | – | – | – | 0.95 (0.55–1.62) | 0.844 |

^a^Total number of follow-up observations included in each model. Each observation corresponds to one follow-up assessment (FU1 or FU2) per participant.

^b^Variance component of the participant-level random intercept.

Notes. Odds ratios (ORs) with 95% confidence intervals (CIs) from logistic mixed-effects models with a random intercept at participant level (binomial distribution, logit link). Model 1 includes time only. Model 2 is adjusted for age, gender, DHI score, multimorbidity, education, and marital status. Model 3 additionally includes self-efficacy, health-related risk attitudes, and future- and present-oriented time preferences. Time is coded as FU2 compared with FU1. Reference categories are female gender, no multimorbidity, low education, and single marital status. Continuous covariates were mean-centred. Models 1 and 2 used complete cases for the variables included in those models, whereas Model 3 used complete cases for all covariates in the full model; therefore, the number of observations differs between models. Analyses were restricted to participants who reported receiving a recommendation for specific exercises and provided at least one valid adherence rating. DHI Dizziness Handicap Inventory, FU1 first follow-up, FU2 second follow-up.

**Supplementary Table S5.** Associations of baseline factors and follow-up time with adherence to lifestyle-related recommendations: sequential logistic mixed-effects models with participant-level random intercepts

| **Predictor** | **Model 1**  **OR (95% CI)** | **p** | **Model 2**  **OR (95% CI)** | **p** | **Model 3**  **OR (95% CI)** | **p** |
| --- | --- | --- | --- | --- | --- | --- |
| N (observations)^a^ | 354 |  | 354 |  | 342 |  |
| Between-person variance^b^ | 2.118 |  | 2.046 |  | 2.076 |  |
| Time (12 months vs. 3 months) | 0.77 (0.44–1.36) | 0.368 | 0.76 (0.43–1.34) | 0.344 | 0.70 (0.39–1.27) | 0.242 |
| Age | – | – | 0.98 (0.94–1.03) | 0.447 | 1.00 (0.95–1.05) | 0.847 |
| Gender (ref = Female) |  |  |  |  |  |  |
| Male | – | – | 0.49 (0.22–1.08) | 0.076 | 0.37 (0.16–0.88) | 0.025 |
| DHI score | – | – | 0.99 (0.98–1.01) | 0.416 | 1.00 (0.98–1.02) | 0.910 |
| Multimorbidity (ref = No) | | – |  |  |  |  |
| Yes | – | – | 1.09 (0.53–2.24) | 0.812 | 1.04 (0.49–2.20) | 0.928 |
| Education (ref = Low) | |  |  |  |  |  |
| Middle | | – | 1.46 (0.59–3.60) | 0.410 | 1.33 (0.52–3.39) | 0.551 |
| High | – | – | 1.81 (0.77–4.28) | 0.176 | 1.34 (0.54–3.34) | 0.525 |
| Marital status (ref = Single) | |  |  |  |  |  |
| Married | – | – | 1.86 (0.43–8.13) | 0.407 | 2.28 (0.50–10.50) | 0.289 |
| Divorced | – | – | 1.23 (0.23–6.52) | 0.808 | 1.16 (0.20–6.88) | 0.867 |
| Widowed | – | – | 1.68 (0.27–10.34) | 0.573 | 1.90 (0.29–12.45) | 0.505 |
| Self-efficacy | – | – | – | – | 1.65 (1.02–2.66) | 0.042 |
| Health-related risk attitudes | – | – | – | – | 0.89 (0.75–1.06) | 0.182 |
| Future-oriented time preference | – | – | – | – | 1.16 (0.96–1.41) | 0.120 |
| Present-oriented time preference | – | – | – | – | 0.85 (0.58–1.25) | 0.401 |

^a^Total number of follow-up observations included in each model. Each observation corresponds to one follow-up assessment (FU1 or FU2) per participant.

^b^Variance component of the participant-level random intercept.

Notes. Odds ratios (ORs) with 95% confidence intervals (CIs) from logistic mixed-effects models with a random intercept at participant level (binomial distribution, logit link). Model 1 includes time only. Model 2 is adjusted for age, gender, DHI score, multimorbidity, education, and marital status. Model 3 additionally includes self-efficacy, health-related risk attitudes, and future- and present-oriented time preferences. Time is coded as FU2 compared with FU1. Reference categories are female gender, no multimorbidity, low education, and single marital status. Continuous covariates were mean-centred. Models 1 and 2 used complete cases for the variables included in those models, whereas Model 3 used complete cases for all covariates in the full model; therefore, the number of observations differs between models. Analyses were restricted to participants who reported receiving at least one lifestyle-related recommendation and provided at least one valid adherence rating. DHI Dizziness Handicap Inventory, FU1 first follow-up, FU2 second follow-up.

**Supplementary Table S6.** Sensitivity analyses for adherence to specific exercises over follow-up (FU1–FU2) using an alternative multimorbidity definition or a stricter adherence cut-off

| **Predictor** | **Model S1a OR (95% CI)** | **p** | **Model S1b OR (95% CI)** | **p** |
| --- | --- | --- | --- | --- |
| N (observations)^a^ | 303 |  | 303 |  |
| Between-person variance^b^ | 4.805 |  | 6.370 |  |
| Time (12 months vs. 3 months) | 0.29 (0.12–0.67) | 0.004 | 0.57 (0.27–1.20) | 0.138 |
| Age | 0.96 (0.90–1.04) | 0.333 | 0.94 (0.87–1.03) | 0.173 |
| Gender (ref = Female) |  |  |  |  |
| Male | 0.79 (0.26–2.38) | 0.670 | 1.54 (0.45–5.29) | 0.494 |
| DHI score | 1.03 (1.00–1.06) | 0.028 | 1.02 (0.99–1.05) | 0.164 |
| Multimorbidity (ref = No) | 2.21 (0.69–7.08) | 0.182 | 0.22 (0.06–0.81) | 0.023 |
| Education (ref = Low) | |  |  |  |
| Middle | 2.42 (0.62–9.44) | 0.202 | 2.24 (0.51–9.91) | 0.286 |
| High | 2.41 (0.64–9.07) | 0.192 | 2.07 (0.50–8.63) | 0.319 |
| Marital status (ref = Single) | |  |  |  |
| Married | 1.96 (0.21–18.29) | 0.556 | 2.37 (0.19–29.54) | 0.503 |
| Divorced | 1.13 (0.09–13.87) | 0.926 | 0.69 (0.04–11.93) | 0.796 |
| Widowed | 0.86 (0.06–12.47) | 0.910 | 2.45 (0.11–54.83) | 0.573 |
| Self-efficacy | 1.37 (0.69–2.69) | 0.369 | 3.01 (1.20–7.59) | 0.019 |
| Health-related risk attitudes | 1.05 (0.83–1.34) | 0.667 | 0.89 (0.68–1.17) | 0.398 |
| Future-oriented time preference | 1.29 (0.99–1.70) | 0.061 | 1.14 (0.86–1.52) | 0.347 |
| Present-oriented time preference | 0.97 (0.57–1.65) | 0.909 | 1.04 (0.59–1.84) | 0.881 |

^a^Total number of follow-up observations included in each model. Each observation corresponds to one follow-up assessment (FU1 or FU2) per participant.

^b^Variance component of the participant-level random intercept.

Notes. Odds ratios (ORs) with 95% confidence intervals (CIs) from logistic mixed-effects models with a random intercept at participant level (binomial distribution, logit link). Both models used the same multivariable model structure as the main analysis, with modified specifications. Model S1a applies an alternative definition of multimorbidity (≥3 chronic conditions). Model S1b applies a stricter adherence definition (Likert scores 3–4 vs. 0–2). Time is coded as FU2 compared with FU1. Reference categories are female gender, no multimorbidity (according to the respective definition), low education, and single marital status. Continuous covariates were mean-centred. Analyses were restricted to participants who reported receiving a recommendation for specific exercises and provided at least one valid adherence rating. DHI Dizziness Handicap Inventory, FU1 first follow-up, FU2 second follow-up.

**Supplementary Table S7.** Sensitivity analyses for adherence to lifestyle-related recommendations over follow-up (FU1–FU2) using an alternative multimorbidity definition or a less stringent adherence cut-off

| **Predictor** | **Model S2a OR (95% CI)** | **p** | **Model S2b OR (95% CI)** | **p** |
| --- | --- | --- | --- | --- |
| N (observations)^a^ | 342 |  | 342 |  |
| Between-person variance^b^ | 2.008 |  | 57.848 |  |
| Time (12 months vs. 3 months) | 0.70 (0.39–1.27) | 0.244 | 0.93 (0.20–4.37) | 0.930 |
| Age | 0.99 (0.95–1.04) | 0.782 | 0.99 (0.83–1.19) | 0.938 |
| Gender (ref = Female) |  |  |  |  |
| Male | 0.37 (0.16–0.87) | 0.022 | 0.19 (0.01–6.11) | 0.352 |
| DHI score | 1.00 (0.98–1.02) | 0.905 | 0.99 (0.93–1.06) | 0.874 |
| Multimorbidity (ref = No) | 1.57 (0.69–3.56) | 0.284 | 0.68 (0.04–10.90) | 0.787 |
| Education (ref = Low) |  |  |  |  |
| Middle | 1.32 (0.52–3.34) | 0.558 | 1.34 (0.05–37.03) | 0.862 |
| High | 1.40 (0.56–3.45) | 0.470 | 1.12 (0.04–28.40) | 0.944 |
| Marital status (ref = Single) | |  |  |  |
| Married | 2.27 (0.50–10.25) | 0.288 | 17.45 (0.12–2646.41) | 0.264 |
| Divorced | 1.15 (0.20–6.67) | 0.877 | 28.09 (0.04–20272.58) | 0.321 |
| Widowed | 1.79 (0.28–11.56) | 0.540 | 4.26 (0.02–1033.74) | 0.605 |
| Self-efficacy | 1.69 (1.04–2.75) | 0.033 | 0.66 (0.12–3.65) | 0.630 |
| Health-related risk attitudes | 0.88 (0.75–1.05) | 0.155 | 0.94 (0.53–1.69) | 0.844 |
| Future-oriented time preference | 1.17 (0.96–1.41) | 0.112 | 1.27 (0.65–2.49) | 0.482 |
| Present-oriented time preference | 0.87 (0.59–1.27) | 0.468 | 0.46 (0.10–2.05) | 0.309 |

^a^Total number of follow-up observations included in each model. Each observation corresponds to one follow-up assessment at FU1 or FU2 per participant.

^b^Variance component of the participant-level random intercept.

Notes. Odds ratios (ORs) with 95% confidence intervals (CIs) from logistic mixed-effects models with a random intercept at participant level (binomial distribution, logit link). Both models used the same multivariable model structure as the main analysis, with modified specifications. Model S2a applies an alternative definition of multimorbidity (≥3 chronic conditions). Model S2b applies a less stringent adherence definition (Likert scores 2–4 vs. 0–1). Time is coded as FU2 compared with FU1. Reference categories are female gender, no multimorbidity (according to the respective definition), low education, and single marital status. Continuous covariates were mean-centred. Analyses were restricted to participants who reported receiving at least one lifestyle-related recommendation and provided at least one valid adherence rating. DHI Dizziness Handicap Inventory, FU1 first follow-up, FU2 second follow-up.
